# Morphokinetic parameters of mouse oocyte meiotic maturation and cumulus expansion are not affected by reproductive age or ploidy status

**DOI:** 10.1007/s10815-023-02779-y

**Published:** 2023-04-04

**Authors:** Chanakarn Suebthawinkul, Elnur Babayev, Hoi Chang Lee, Francesca E. Duncan

**Affiliations:** 1 Department of Obstetrics and Gynecology, Feinberg School of Medicine, Northwestern University, Chicago, IL USA; 2 Department of Obstetrics and Gynecology, Faculty of Medicine, Chulalongkorn University, Bangkok, Thailand

**Keywords:** In vitro maturation, Time-lapse, Meiotic progression, Cumulus expansion, Morphokinetics, Reproductive aging

## Abstract

**Introduction:**

Morphokinetic analysis using a closed time-lapse monitoring system (EmbryoScope + ™) provides quantitative metrics of meiotic progression and cumulus expansion. The goal of this study was to use a physiologic aging mouse model, in which egg aneuploidy levels increase, to determine whether there are age-dependent differences in morphokinetic parameters of oocyte maturation.

**Methods:**

Denuded oocytes and intact cumulus-oocyte complexes (COCs) were isolated from reproductively young and old mice and in vitro matured in the EmbryoScope + ™. Morphokinetic parameters of meiotic progression and cumulus expansion were evaluated, compared between reproductively young and old mice, and correlated with egg ploidy status.

**Results:**

Oocytes from reproductively old mice were smaller than young counterparts in terms of GV area (446.42 ± 4.15 vs. 416.79 ± 5.24 µm^2^, *p* < 0.0001) and oocyte area (4195.71 ± 33.10 vs. 4081.62 ± 41.04 µm^2^, *p* < 0.05). In addition, the aneuploidy incidence was higher in eggs with advanced reproductive age (24–27% vs. 8–9%, *p* < 0.05). There were no differences in the morphokinetic parameters of oocyte maturation between oocytes from reproductively young and old mice with respect to time to germinal vesicle breakdown (GVBD) (1.03 ± 0.03 vs. 1.01 ± 0.04 h), polar body extrusion (PBE) (8.56 ± 0.11 vs. 8.52 ± 0.15 h), duration of meiosis I (7.58 ± 0.10 vs. 7.48 ± 0.11 h), and kinetics of cumulus expansion (0.093 ± 0.002 vs. 0.089 ± 0.003 µm/min). All morphokinetic parameters of oocyte maturation were similar between euploid and aneuploid eggs irrespective of age.

**Conclusion:**

There is no association between age or ploidy and the morphokinetics of mouse oocyte in vitro maturation (IVM). Future studies are needed to evaluate whether there is an association between morphokinetic dynamics of mouse IVM and embryo developmental competence.

**Supplementary Information:**

The online version contains supplementary material available at 10.1007/s10815-023-02779-y.

## Introduction

Over the past decade, time-lapse technology has been widely used in clinical Assisted Reproductive Technology (ART) laboratories to monitor human preimplantation embryo development and to develop predictive algorithms for non-invasive embryo assessment and selection [1–3]. Closed time-lapse imaging incubators provide an uninterrupted optimal culture environment by maintaining a steady temperature, oxygen concentration, and humidity, which may mimic the in vivo environment better than traditional incubators [3–5]. Moreover, time-lapse systems provide a vast amount of data regarding time-specific morphological changes in the preimplantation embryo, enabling a non-invasive and comprehensive view of early development [6–10]. However, the clear clinical benefit of the use of time-lapse technology to improve ART outcomes remains to be proven [11].

Although morphokinetic analysis using closed time-lapse monitoring systems provides powerful quantitative metrics regarding biological processes, it has primarily been limited in the field of reproductive science and medicine to preimplantation embryos. We recently, however, extended the use of this technology to oocyte maturation using a mouse model [12]. Oocyte maturation is a complex sequence of nuclear and cytoplasmic events that occur in parallel with changes in the surrounding cumulus cells to prepare oocytes for successful fertilization and embryo development [13, 14]. In response to the luteinizing hormone (LH) surge, several signaling pathways are initiated leading to the resumption and progression of meiosis. The oocyte transitions from the diakinesis stage of prophase of meiosis I to metaphase of meiosis II (MII). This process is characterized by nuclear (germinal vesicle) envelope breakdown (GVBD), meiotic spindle assembly, rearrangement of the cortical cytoskeleton, and extrusion of the first polar body (PBI) [14–16]. Alongside meiotic maturation of the oocyte, surrounding cumulus cells undergo maturation-associated changes. The LH surge induces cumulus layer expansion through increased synthesis and accumulation of hyaluronan (HA) and the associated extracellular protein matrix [17–20]. Cumulus expansion facilitates the developmental competence of the resulting gamete and follicle rupture upon ovulation and also plays an important role in fertilization [21–23]. Therefore, both meiotic progression and cumulus expansion are involved in the acquisition of oocyte developmental competence [24].

Oocyte meiotic maturation and cumulus expansion can be recapitulated in vitro in both cumulus oocyte complexes (COCs) and denuded oocytes devoid of cumulus cells [25]. Morphological hallmarks that can be visually observed include nuclear meiotic maturation and cumulus expansion. The loss of germinal vesicle (GV) denotes the transition of arrested prophase I oocyte to meiosis. The extrusion of PBI indicates completion of meiosis I and the transition of the oocyte to meiosis II [13, 14]. These cellular features identify the precise timing of the cell cycle, and alterations in these processes lead to detrimental effects on oocyte quality [26, 27]. Simultaneously, cumulus expansion can be visualized as a transition from compact cumulus cell layers into a dispersed structure of cells as a result of cellular proliferation combined with synthesis and accumulation of extracellular matrix [20]. Perturbation in cumulus expansion impairs oocyte meiotic maturation, ovulation, fertilization, and embryo development [28, 29].

We recently established reproducible baseline morphokinetic parameters of mouse oocyte in vitro maturation (IVM) and identified novel dynamics of oocyte meiotic maturation and cumulus expansion using a closed time-lapse incubator system [12]. Furthermore, we validated these established parameters by demonstrating their sensitivity to known perturbations of meiotic maturation and cumulus expansion. Thus, being able to correlate morphokinetic parameters of IVM with outcomes such as egg aneuploidy and developmental competence may have clinical relevance as a non-invasive indicator of gamete quality. In our initial study, the morphokinetic parameters of meiotic maturation were similar between euploid and aneuploid eggs, but our sample size was small because we used reproductively young mice where the natural incidence of aneuploidy is low [12].

Therefore, the goal of this study was to extend our initial findings to a physiologic aging mouse model where egg quality is inherently reduced and aneuploidy levels are higher [30]. Using the EmbryoScope + ™ platform (Vitrolife, Denver, CO), we examined the relationship between advanced reproductive age and egg ploidy status on the morphological and morphokinetic parameters of IVM. Despite the GV and oocyte area being significantly decreased in oocytes from reproductively old mice relative to young counterparts, no age- or ploidy-dependent differences in morphokinetic parameters of oocyte meiotic maturation were observed. Overall these findings demonstrate that morphokinetic parameters of IVM are not associated with oocyte meiotic competence but further studies are needed to determine how they relate to embryo developmental competence and whether they can be harnessed in the clinical ART setting.

## Materials and methods

### Animals

Reproductively young (6–12 week old) and old (13–15 month old) CD1 female mice were obtained from Envigo (Indianapolis, IN). Based on a linear extrapolation of age, the reproductively young mice are equivalent to women in their 20 s, whereas the reproductively old cohort corresponds to women in their late thirties to early forties [31, 32]. Mice were housed in a controlled barrier facility at Northwestern University’s Center for Comparative Medicine in Chicago under constant temperature, humidity, and light (14 h light/10 h dark). Mice were provided food and water ad libitum. All animal experiments described were approved by the Institutional Animal Care and Use Committee (Northwestern University) and performed under the National Institutes of Health Guidelines.

### Ovarian hyperstimulation and COC collection

To maximize the yield of COCs collected, reproductively young and old mice were stimulated with intraperitoneal (IP) injections of 5 IU pregnant mare serum gonadotropin (PMSG) (ProSpec-Tany TechnoGene, East Brunswick NJ, Cat # HOR-272), and 44–46 h post-PMSG injection, ovaries were harvested. Isolated ovaries were placed into dishes containing pre-warmed Leibovitz's medium (L15) (Life Technologies Corporation, Grand Island, NY) supplemented with 3 mg/ml polyvinylpyrrolidone (PVP) (Sigma-Aldrich, St. Louis, MO) and 0.5% (v/v) Penicillin–Streptomycin (PS) (Life Technologies Corporation, Grand Island, NY) (L15/PVP/PS)*.* Antral follicles were mechanically punctured with insulin syringes to release COCs from the ovaries. COCs were transferred to L15/PVP/PS medium containing 2.5 μM milrinone (Sigma-Aldrich, St. Louis, MO), a PDE3A inhibitor that maintains oocytes arrested in prophase I [33]. To obtain denuded oocytes, the surrounding cumulus cells were removed from the COCs by mechanical disruption. The resulting denuded oocytes were allowed to recover in α-MEM + GlutaMAX (Thermo Fisher Scientific, Waltham, MA)/PS/ Bovine Serum Albumin (BSA) (Sigma-Aldrich, St. Louis, MO) (α-MEM/PS/BSA) supplemented with 2.5 μM milrinone for 1 h at 37^0^C in a humidified atmosphere of 5% CO2 in air prior to being loaded into an EmbryoSlide (Vitrolife, Denver, CO). At least 3 independent replicates were performed for each experiment. The COCs or denuded oocytes were pooled together from 2—4 animals per age group per experiment to minimize any animal-specific variability. Denuded oocytes or COCs from reproductively young and old mice were in vitro matured in parallel and treated similarly in all experiments.

### In vitro maturation within the EmbryoSlide in EmbryoScope + ™

The 16 microwells in the EmbryoSlides (Vitrolife, Denver, CO), each with a diameter of approximately 250 μm, were filled according to the manufacturer’s instructions with the specific maturation medium designated for oocytes or COCs as described below. The microwells and wells were overlaid with 1.6 mL of mineral oil (Sigma-Aldrich, St. Louis, MO) and equilibrated in the EmbryoScope + ™ for 9–24 h [12].

Oocyte maturation was induced by the removal of milrinone, which results in the degradation of cAMP and synchronous spontaneous meiotic resumption of the oocyte [13, 34]. Depending on the experiment, denuded oocytes or COCs were loaded into the wells of the EmbryoSlide containing pre-equilibrated medium. Denuded oocytes were matured in α-MEM/PS/BSA medium, whereas the intact COCs were matured in specific medium that induces and supports cumulus expansion (α-MEM Glutamax supplemented with 5%(v/v) Fetal bovine serum (FBS) / 0.02%(v/v) Epidermal growth factor (EGF)/ 20 mM HEPES/ 0.25 mM pyruvate) [35, 36]. EGF, HEPES, and pyruvate were purchased from Sigma-Aldrich, St. Louis, MO, and FBS was purchased from Thermo Fisher Scientific, Waltham, MA. EmbryoSlides were then loaded into the EmbryoScope + ™ [12]. Denuded oocytes or COCs were in vitro matured for a total of 16 h at 37^0^C in a humidified atmosphere of 5% CO2 in air. Images were taken every 10 min at 11 focal planes with low-intensity red LED illumination with < 0.5 s of light exposure per image. These conditions are identical to those used for humans in ART, and therefore, are considered to have minimal impact (if any) on gametes and preimplantation embryos. This technology can accommodate simultaneous and continuous monitoring of 240 samples and eliminates the need to image outside of the incubator.

After IVM, the meiotic maturation status of each oocyte was assessed based on morphological criteria. For in vitro matured COCs, the surrounding cumulus cells were removed following a brief incubation in 0.25 mg/ml hyaluronidase (Sigma-Aldrich, St. Louis, MO) so that the meiotic stage of the oocyte could be accurately visualized. Oocytes that failed to mature and remained arrested at prophase of meiosis I were characterized by an intact nucleus and considered germinal vesicle-intact (GV). Oocytes that lacked a nucleus but had not yet extruded the first polar body were considered to have undergone germinal vesicle breakdown (GVBD). Cells that had extruded the first polar body (PBE) were considered mature. The percentage of oocytes at each stage (GV, GVBD, and PBE) was reported in all experiments.

### Analysis of timelapse data for denuded oocytes

**Fig. 1:**
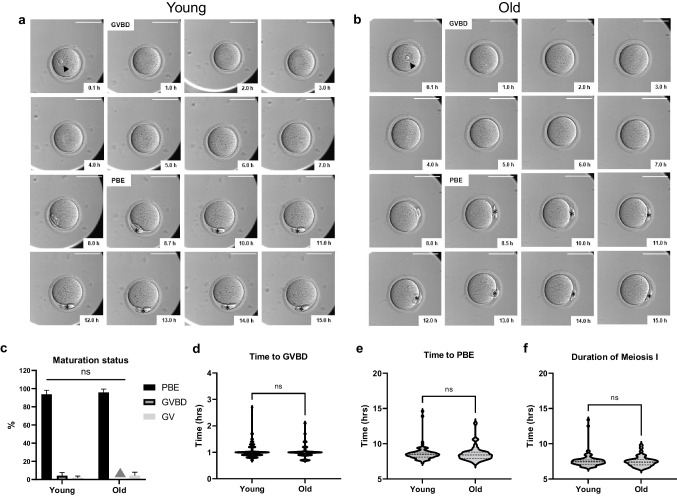
Baseline morphokinetic parameters of meiotic progression in reproductively young and old mouse denuded oocytes during IVM. (**a**-**b**) A representative series of montage images show meiotic progression in an individual young and old oocyte within the EmbryoScope + ™. The time when denuded oocytes were put into the EmbryoScope + ™ was set as the starting point. Time to GVBD referred to the first time that we did not observe the germinal vesicle membrane (1.0 h in both young and old oocytes), and time to PBE represented the first time when PBI membrane completely separated from the oocyte membrane (8.7 h in young oocyte and 8.5 h in old oocyte). Time difference between GVBD and PBE is the duration of meiosis I which is 7.7 h and 7.5 h in young and old oocytes respectively. (Scale bar = 100 µm) (**c**) Maturation status of young and old denuded oocytes after IVM (grey triangle indicates no oocytes in old GVBD group). (**d**-**f**) Morphokinetic parameters of meiotic progression between young and old oocytes including (**d**) time to GVBD, (**e**) time to PBE, and (**f**) duration of meiosis I. (*n* = 96 for young, *n* = 47 for old, 3 replicates) GVBD; germinal vesicle breakdown, PBE; polar body extrusion, MI; meiosis I, PBI, first polar body, IVM; in vitro maturation, arrowhead; GV, asterisk; PBI

**Fig. 2:**
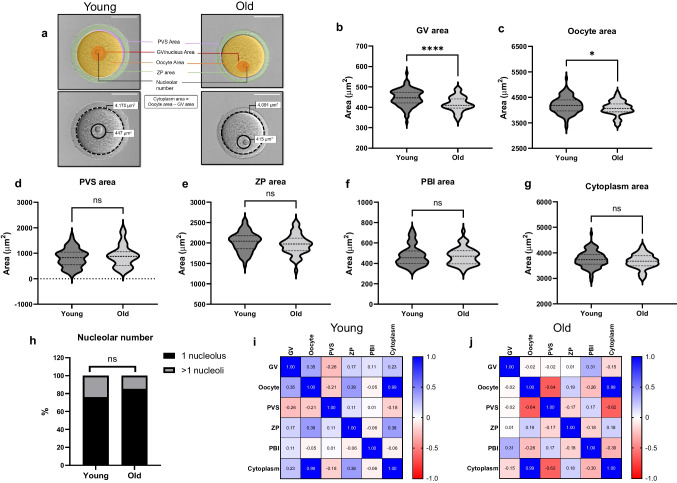
Morphological parameters (**a**) of the young and old denuded oocytes. Cytoplasm area was calculated by subtracting the GV area from the oocyte area. Lower panel shows the representative images of young and old oocytes labeled with the oocyte area (dotted line) and GV area (solid line) (Scale bar = 50 µm). (**b**-**h**) The comparison of morphological parameters between young and old mouse denuded oocytes. (**i**-**j**) The matrix shows correlation (r-value) between each morphological parameter in (**i**) young and (**j**) old oocytes. Blue color represents a positive correlation. Red color represents a negative correlation. (*n* = 96 for young, *n* = 47 for old, 3 replicates) (ns; *p* > 0.05, *; *p* < 0.05, ****; *p* < 0.0001). GV; Germinal vesicle, PVS; perivitelline space, ZP; zona pellucida, PBI; first polar body

For evaluation of morphological and morphokinetic parameters of meiotic progression, denuded oocytes from reproductively young (*n* = 96 oocytes, 6 mice, 3 replicates) and old (*n* = 47 oocytes, 6 mice, 3 replicates) mice were matured in the EmbryoScope + ™ (Supplemental Video 1). The timelapse imaging data were evaluated using analysis software provided by the manufacturer (EmbryoViewer, Vitrolife, Denver, MO) which includes an annotation function to capture information and is intended for displaying, storing, and transferring images generated by the EmbryoScope + ™. The morphokinetic parameters of meiotic progression, including time to GVBD, time to first polar body extrusion (PBE), and duration of meiosis I were determined following IVM of denuded oocytes. The time when denuded oocytes were placed into the EmbryoScope + ™ was set as the starting point. The time to GVBD was defined as the first time when the loss of the GV was observed, and the time to PBE was defined when cytokinesis was complete and the PBI membrane was completely separated from the oocyte plasma membrane rather than the beginning of PBI extrusion (Fig. 1a-b). Although Meiosis I is initiated during fetal life, in this study we define the duration of Meiosis I in reference to the process of meiotic maturation as the time difference between GVBD and PBE [7, 12, 37–39]. In addition to the morphokinetics, we also assessed other morphological parameters of denuded oocytes using the EmbryoViewer. There were 11 images taken at every time point through the z-axis. Thus, images were reviewed and the focal plane where the structure of interest was best in focus was used for analysis. For example, to obtain accurate measurements of the oocyte diameter, the plasma membrane of the oocyte was in focus. To obtain accurate measurements of the GV diameter, the nuclear envelope was in focus. For any given oocyte, the focal plane in which the plasma membrane was in focus may have been different from that in which the nuclear envelope was in focus. The annotation function in the software was used to demarcate the structure and the area measurement for this region of interest was recorded (Fig. 2a) [12, 40–45]. These parameters included: GV or nucleus area, oocyte area, perivitelline space (PVS) area, zona pellucida (ZP) area, cytoplasm area, and nucleolar number of individual oocytes which were assessed at the beginning of IVM. The cytoplasm area was calculated by subtracting the GV area from the oocyte area. The PBI area was assessed at the end of IVM [12].

### Analysis of timelapse data for intact COCs

For evaluation of morphokinetic parameters of cumulus layer expansion, COCs from reproductively young (*n* = 80 COCs, 6 mice, 3 replicates) and old (*n* = 44 COCs, 6 mice, 3 replicates) mice were matured in the EmbryoScope + ™ (Supplemental Video 2). After IVM of COCs, morphokinetic parameters of cumulus layer expansion were evaluated with the EmbryoViewer [12]. The time when COCs were placed into the EmbryoScope + ™ was set as the starting point. The distance of cumulus layer expansion was measured every 1 h at the same position until the end of expansion or until the cumulus layer expanded beyond the well limits. The position where COCs had the widest space to expand was selected to conduct the measurements to minimize the limitation of the expansion beyond the well. The overall rate of cumulus layer expansion, the velocity of cumulus expansion at every 1 h, and the velocity of cumulus expansion at every 4 h were calculated by using these formulas:

$$Overall\;rate\;of\;expansion\;\left(\mu m/min\right)=\frac{Distance\;at\;the\;end\;of\;expansion-Distance\;at\;start}{Time\;at\;the\;end\;of\;expansion-Time\;at\;start}$$

$$Velocity\;of\;expansion\;at\;each\;time\;point\;\left(\mu m/min\right)=\frac{Distance\;at\;2nd\;time\;point-Distance\;at\;1st\;time\;point}{Time\;at\;2nd\;point-Time\;at\;1st\;point}$$

### Ploidy analysis

After IVM, the resulting cells that had undergone PBE were evaluated for ploidy status using the in-situ chromosome spreading method [12, 46]. All oocytes were stained and tracked individually throughout the experiment so that the ploidy data could be directly correlated with the morphokinetic and morphological parameters. MII eggs were first treated with 100 µM Monastrol (Tocris Bioscience, Bristol, UK) which collapses the bipolar spindle into a monopolar one and results in the dispersion of the chromosomes within an intact cell [47]. This incubation was performed at 37 °C in a humidified atmosphere of 5% CO2 in air for 3 h. The eggs were fixed in 2% paraformaldehyde (Electron Microscopy Sciences, Hatfield, PA) for 20 min at room temperature. After fixation, the eggs were washed with the blocking buffer (1X PBS, 0.01% Tween-20 (Sigma-Aldrich, St. Louis, MO), 0.02% sodium azide (NaN3) (Sigma-Aldrich, St. Louis, MO), and 0.3% BSA) twice for 5 min. Then they were treated with the permeabilization solution (1X PBS, 0.1% TX-100, 0.02% NaN3, and 0.3% BSA) for 15 min at room temperature and were washed again with the blocking buffer. To detect kinetochores, the eggs were incubated with the primary antibody (1:200 human anti-centromere/kinetochore, Antibodies Incorporated, Davis, CA, Cat # 15–234) at 4 °C overnight. The cells were then rinsed with the blocking buffer three times for 20 min and incubated with the secondary antibody (1:100, goat anti-human IgG (H + L) AlexaFluor 488, Invitrogen, Waltham, MA, Cat # A-11013) for 1 h at room temperature. Then the eggs were washed again with the blocking buffer three times and mounted in Vectashield Antifade Mounting Medium with DAPI (4’,6-diamidino-2-phenylindole; Vector Laboratories, Burlingame, CA). Eggs were imaged on a Leica SP5 inverted laser scanning confocal microscope using 405 nm and 488 nm lasers (Leica Microsystems, Wetzlar, Germany). For the kinetochore analysis, the imaging was performed under 100 × magnification and Z-stack thickness was 0.5 µm [48]. Ploidy status was evaluated by manually counting the kinetochores in each z-plane through a stack encompassing the entire oocyte. Two investigators blinded to the experimental conditions performed the counting [49]. A euploid mouse egg contains a total of 20 pairs of sister chromatids with 40 kinetochores, and any egg that differed from these numbers was considered aneuploid. All images were processed using LAS AF (Leica Microsystems, Wetzlar, Germany) and analyzed using FIJI (National Institutes of Health, Bethesda, MD).

### Statistical analysis

Data are presented as the mean ± SEM or percentage of proportion (%), and each experiment was repeated at least three times. All results were graphed using GraphPad Prism Software Version 9.3.1 (La Jolla, California). The normal distribution of data was evaluated with the Shapiro–Wilk test. Analysis between groups of continuous variables were performed with students’ t-test or Mann–Whitney U test. Multiple comparisons were analyzed with one-way ANOVA test, Kruskal–Wallis test, and two-way ANOVA (mixed-effects analysis) followed by Tukey’s multiple-comparison tests. Categorical variables were analyzed with Fisher's exact test or Chi-square test. The correlation between continuous variables was analyzed with the Pearson Correlation test. *P* values < 0.05 were considered statistically significant.

## Results

### Morphokinetic parameters of meiotic progression are similar in oocytes from reproductively young and old mice

To determine whether there are reproductive age-dependent differences in morphokinetic parameters of meiotic progression, we matured denuded oocytes from reproductively young and old mice in the EmbryoScope + ™ (Fig. 1a-b, Supplemental Video 1). 93.75 ± 3.22% of denuded oocytes from reproductively young mice underwent PBE within the EmbryoScope + ™, whereas 2.09 ± 1.04% remained arrested in prophase I (GV) and 4.17 ± 2.08% were either in pro-metaphase I or metaphase I (GVBD) (Table 1, Fig. 1c). For reproductively old mice, 95.6 ± 2.16% of denuded oocytes reached PBE, while the rest (4.31 ± 2.16%) remained in GV stage. The ability to undergo PBE did not differ between the young (93.75 ± 3.22%) and old groups (95.6 ± 2.16%) (*p* > 0.05, Table 1, Fig. 1c). Among the oocytes that emitted a polar body, there were no age-dependent differences in time to GVBD (1.03 ± 0.03 vs. 1.01 ± 0.04 h), time to PBE (8.56 ± 0.11 vs. 8.52 ± 0.15 h), or duration of meiosis I (7.58 ± 0.10 vs. 7.48 ± 0.11 h) (*p* > 0.05, Table 1, Fig. 1d-f).

**Table 1:** Comparisons of baseline parameters of reproductively young and old mouse denuded oocytes and COCs during IVM in closed time-lapse incubator (Mean ± SEM)

Denuded Oocytes			
Parameters	Young (*n *= 96, 6 mice)	Old (*n* = 47, 6 mice)	*P* value
PBE rate (%)	93.75 ± 3.22%	95.60 ± 2.16%	> 0.999
Euploidy rate	91.10 ± 2.10%	76.11 ± 2.00%	0.031
Morphological parameters			
Nucleolar number			
1 nucleolus	76.04%	85.11%	0.073
> 1 nucleolus	23.96%	14.89%	
GV area (µm^2^)	446.42 ± 4.15	416.79 ± 5.24	< 0.0001
Oocyte area (µm^2^)	4195.71 ± 33.10	4081.62 ± 41.04	0.041
PVS area (µm^2^)	820.11 ± 41.20	895.63 ± 63.58	0.301
ZP area (µm^2^)	2024.49 ± 24.36	1965.49 ± 36.06	0.172
PBI area (µm^2^)	473.26 ± 10.40	470.67 ± 12.68	0.881
Cytoplasm area (µm^2^)	3749.29 ± 32.00	3664.83 ± 41.50	0.121
Morphokinetic parameters			
Time to GVBD (hr)	1.03 ± 0.03	1.01 ± 0.04	0.942
Time to PBE (hr)	8.56 ± 0.11	8.52 ± 0.15	0.387
Duration of Meiosis I (hr)	7.58 ± 0.10	7.48 ± 0.11	0.604
Cumulus oocyte complexes			
Parameters	Young (*n* = 80, 6 mice)	Old (*n* = 44, 6 mice)	*P* value
Maturation rate	97.50 ± 1.53%	94.44 ± 5.56%	0.599
Euploidy rate	92.31 ± 1.07%	72.72 ± 4.49%	0.031
Overall rate of cumulus expansion (µm/min)	0.093 ± 0.002	0.089 ± 0.003	0.350

IVM; in vitro maturation, GV; Germinal vesicle, PVS; perivitelline space, ZP; zona pellucida, PBI; first polar body, GVBD; germinal vesicle breakdown, PBE; polar body extrusion, COCs; cumulus-oocyte complexes, SEM; standard error of the mean

### GV and oocyte area are significantly smaller in oocytes of reproductively old mice

In addition to morphokinetic parameters, we evaluated a series of morphological parameters, including GV area, oocyte area, PVS area, ZP area, PBI area, cytoplasm area, and the nucleolar number of individual oocytes from reproductively young and old mice (Fig. 2a). There were no differences in PVS area (820.11 ± 41.20 vs. 895.63 ± 63.58 µm^2^), ZP area (2024.49 ± 24.36 vs. 1965.49 ± 36.06 µm^2^), PBI area (473.26 ± 10.40 vs. 470.67 ± 12.68 µm^2^), and cytoplasm area (3749.29 ± 32.00 vs. 3664.83 ± 41.50 µm^2^) between oocytes from reproductively young and old mice, respectively (*p* > 0.05, Table 1, Fig. 2d-g). However, the GV area (446.42 ± 4.15 vs. 416.79 ± 5.24 µm^2^, *p* < 0.0001) and oocyte area (4195.71 ± 33.10 vs. 4081.62 ± 41.04 µm^2^, *p* = 0.041) were significantly smaller in oocytes from reproductively old mice (Table 1, Fig. 2a-c). The majority of the oocytes in the reproductively young (76.04%) and the old (85.11%) cohorts had one nucleolus. The rest had 2 nucleoli, except for an oocyte in both groups which had 3 nucleoli. We classified the oocytes into 2 groups based on nucleolar number; 1 nucleolus and > 1 nucleoli. There was no difference in nucleolar number between age cohorts (*p* = 0.073, Table 1, Fig. 2h). We analyzed the correlations between the morphological parameters and observed a strong correlation between the cytoplasm and oocyte areas in both age cohorts (r = 0.99, *p* < 0.0001, Fig. 2i-j). We also evaluated the correlation between the morphological and morphokinetic parameters of meiotic progression and did not observe any strong correlations in oocytes from reproductively young or old mice (Supplemental Fig. 1-2).

### Kinetics of cumulus expansion is similar between reproductively young and old mouse COCs

**Fig. 3:**
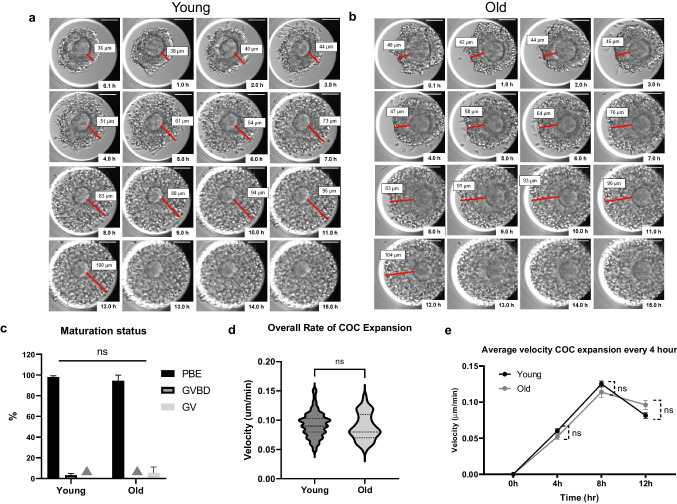
Baseline morphokinetic parameters of cumulus expansion in reproductively young and old mouse COCs during IVM. (**a**-**b**) A representative series of montage images show cumulus layer expansion of an individual young and old COC within the EmbryoScope + ™. The time when COCs were put into the EmbryoScope + ™ was set as the starting point. The distances of cumulus layer expansion (red line) were measured every 1 h at the same position as much as possible until the end of the 16 h observation or until the cumulus layer expanded beyond the well limits. The number of COCs that had fully observable 12-h expansion period were similar in both age groups, 72 out of 80 (90%) in the young group and 39 out of 44 (88.6%) in the old group (*p* = 0.81). (Scale bar = 100 µm) (**c**) Maturation status of young and old COCs after IVM (grey triangle indicates no oocytes in young GV and old MI groups) (**d**-**e**) Morphokinetic parameters of cumulus expansion between young and old mouse COCs, including (**d**) overall rate of cumulus expansion and (**e**) velocity of expansion every 4 h (*n* = 80 for young, *n* = 44 for old, 3 replicates) (ns; *p* > 0.05) COCs; cumulus-oocyte complexes, IVM; in vitro maturation

To determine whether reproductive aging influences morphokinetic parameters of cumulus expansion, we matured the COCs from reproductively young and old mice in the EmbryoScope + ™ (Fig. 3a-b, Supplemental Video 2). We observed that 97.50 ± 1.53% of oocytes within COCs from reproductively young mice progressed to PBE, while 3.13 ± 1.86% were either in pro-metaphase I or metaphase I (GVBD) (Fig. 3c). In reproductively old mice, 94.44 ± 5.56% of oocytes within COCs underwent PBE while the rest (5.56 ± 5.56%) remained in the GV stage (Fig. 3c). The ability to reach PBE did not differ between the reproductively young (97.50 ± 1.53%) and old groups (94.44 ± 5.56%) (*p* > 0.05, Table 1 Fig. 3c). We then evaluated the morphokinetic parameters of cumulus expansion, including the overall rate of cumulus layer expansion, the average velocity of cumulus expansion every 1 h, and the average velocity of cumulus expansion every 4 h. There was no age-dependent difference in the overall rate of cumulus layer expansion (0.093 ± 0.002 in COCs from reproductively young mice vs. 0.089 ± 0.003 µm/min in COCs from reproductively old mice, *p* > 0.05, Table 1, Fig. 3d). The average velocity of cumulus expansion every 1 h and every 4 h were also similar between the groups (*p* > 0.05, Fig. 3e, Supplemental Fig. 3a). The overall dynamics of COC expansion was similar irrespective of age, reaching peak velocity during the first 8 h of maturation and then slowing (Fig. 3e). The timing of the dynamic change in velocity of cumulus layer expansion (at ~ 8 h) correlates with the timing of PBI extrusion (Fig. 1e, Fig. 3e, Supplemental Fig. 3a). However, the kinetics of COC expansion tended to be different with age, with COCs from reproductively young mice expanding faster during the first half of the maturation period and then slowing more rapidly during the second half relative to COCs from reproductively old mice (Fig. 3e).

### Morphokinetic parameters of meiotic progression between euploid and aneuploid eggs are not different regardless of reproductive age

**Fig. 4:**
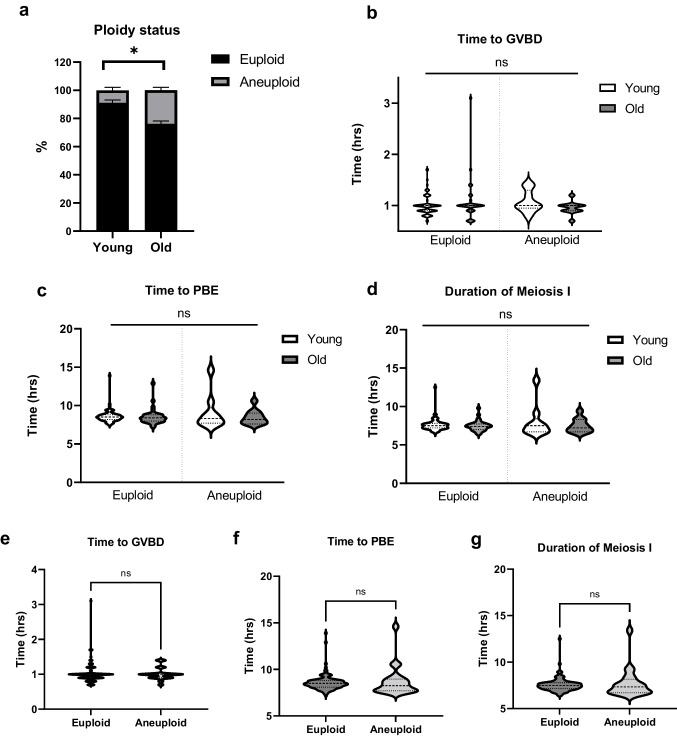
Morphokinetic parameters of meiotic progression during in vitro maturation are similar between euploid and aneuploid eggs regardless of reproductive age. (**a**) Ploidy status of resulting MII eggs between young and old oocytes. (**b**-**d**) Morphokinetic parameters of meiotic progression among 4 groups of eggs (Young-Euploid, Young-Aneuploid, Old-Euploid, and Old-Aneuploid) including (**b**) time to GVBD, (**c**) time to PBE, and (**d**) duration of meiosis I. (**e**–**g**) Morphokinetic parameters between euploid and aneuploid eggs (pooled young and old eggs) including (**e**) time to GVBD, (**f**) time to PBE, and (**g**) duration of meiosis I. (Young; euploid *n* = 81 eggs, aneuploid = 8 eggs, Old; euploid *n* = 33 eggs, aneuploid *n* = 10 eggs, 3 replicates) (ns; *p* > 0.05, *; *p* < 0.05) GVBD; germinal vesicle breakdown, PBE; polar body extrusion

Following IVM of denuded oocytes in EmbryoScope + ™, the incidence of euploid and aneuploid eggs in reproductively young mice was 91.10 ± 2.10% (*n* = 81) and 8.90 ± 2.10% (*n* = 8), respectively, whereas these numbers were 76.11 ± 2.00% (*n* = 33) and 23.92 ± 2.03 (*n* = 10) in reproductively old mice (Table 1, Fig. 4a). The incidence of euploidy was significantly decreased in the reproductively old group (*p* = 0.031). We compared the morphokinetic parameters of meiotic progression among the four cohorts of eggs based on reproductive age and ploidy status; young-euploid (Y-Eu), young-aneuploid (Y-An), old euploid (O-Eu), and old aneuploid (O-An). There were no differences in time to GVBD (Y-Eu; 1.00 ± 0.02 vs. Y-An; 1.08 ± 0.07 h vs. O-Eu; 1.06 ± 0.07 vs. O-An; 0.96 ± 0.04 h), time to PBE (Y-Eu; 8.58 ± 0.09 vs. Y-An; 89.08 ± 0.76 h vs. O-Eu; 8.54 ± 0.28 vs. O-An; 8.46 ± 0.28 h), or duration of meiosis I (Y-Eu; 7.57 ± 0.08 vs. Y-An; 7.51 ± 0.27 h vs. O-Eu; 7.48 ± 0.11 vs. O-An; 7.51 ± 0.27 h) among these groups of eggs (*p* > 0.05, Table 2, Fig. 4b-d). We then pooled the young and old oocytes together and classified them only based on ploidy status. In this overall analysis, no differences were observed in the morphokinetic parameters of meiotic progression including time to GVBD (1.02 ± 0.03 vs. 1.01 ± 0.04 h), time to PBE (8.57 ± 0.08 vs. 8.74 ± 0.37 h), and duration of meiosis I (7.55 ± 0.07 vs. 7.73 ± 0.35 h) between euploid and aneuploid eggs (*p* > 0.05, Fig. 4e-g).

**Table 2:** Morphological and morphokinetic parameters of meiotic progression and cumulus expansion during IVM in closed time-lapse incubator between 4 groups of eggs based on reproductive age and the ploidy status (Mean ± SEM)

Parameters of denuded oocytes*	Euploid		Aneuploid		*P* value
	Young (*n* = 81)	Old (*n* = 33)	Young (*n* = 8)	Old (*n* = 10)	
Morphological parameters					
GV area (µm^2^)	432.18 ± 3.00	417.37 ± 5.55	419.91 ± 5.42	427.45 ± 13.50	0.085
Oocyte area (µm^2^)	4238.12 ± 37.13	4083.72 ± 44.85	4035.29 ± 61.64	4117.64 ± 67.83	0.031
PVS area (µm^2^)	801.94 ± 46.01	897.50 ± 68.39	1024.2 ± 159.90	787.36 ± 60.81	0.372
ZP area (µm^2^)	2060.91 ± 26.98	1978.30 ± 38.14	2061.71 ± 45.78	1993.55 ± 43.29	0.272
PBI area (µm^2^)	476.99 ± 11.76	469.38 ± 11.80	481.29 ± 31.11	426.91 ± 33.79	0.928
Cytoplasm area (µm^2^)	3790.65 ± 35.87	3666.35 ± 45.28	3595.29 ± 62.61	3690.18 ± 74.59	0.205
Morphokinetic parameters					
Time to GVBD (hr)	1.00 ± 0.02	1.06 ± 0.07	1.08 ± 0.07	0.96 ± 0.04	0.500
Time to PBE (hr)	8.58 ± 0.09	8.54 ± 0.28	9.08 ± 0.76	8.46 ± 0.28	0.599
Duration of Meiosis I (hr)	7.57 ± 0.08	7.48 ± 0.11	8.00 ± 0.72	7.51 ± 0.27	0.721
Parameters of COCs**	Euploid		Aneuploid		*P* value
	Young (*n* = 69)	Old (*n* = 28)	Young (*n* = 5)	Old (*n* = 11)	
Overall rate of cumulus expansion (µm/min)	0.091 ± 0.003	0.088 ± 0.004	0.099 ± 0.007	0.108 ± 0.013	0.273

IVM, in vitro maturation, GV, Germinal vesicle, PVS; perivitelline space, ZP, zona pellucida, PBI, first polar body, GVBD, germinal vesicle breakdown, PBE, polar body extrusion, COCs, cumulus-oocyte complexes, SEM, standard error of the mean, *Denuded oocytes from 6 reproductively young mice and 6 reproductively old mice with 3 replicates, **COCs from 6 reproductively young mice and 6 reproductively old mice with 3 replicates

### Morphological parameters of denuded oocytes between euploid and aneuploid eggs are not different regardless of age

**Fig. 5:**
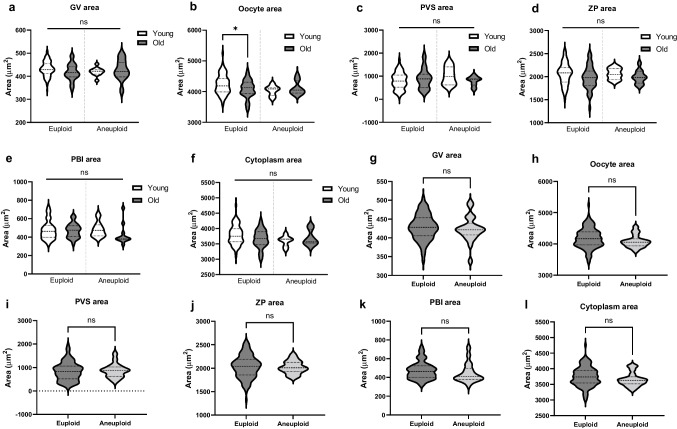
Comparison of oocyte morphological parameters based on reproductive age and ploidy status (**a**-**f**) Morphological parameters among 4 groups of eggs (Young-Euploid, Young-Aneuploid, Old-Euploid, and Old-Aneuploid) (**g**-**l**) Morphological parameters between euploid and aneuploid eggs (pooled young and old eggs) (Young; euploid *n* = 81 eggs, aneuploid = 8 eggs, Old; euploid *n* = 33 eggs, aneuploid *n* = 10 eggs, 3 replicates) (ns; *p* > 0.05, *; *p* < 0.05) GV; Germinal vesicle, PVS; perivitelline space, ZP; zona pellucida, PBI; first polar body

In addition to the morphokinetic parameters, we also compared morphological parameters among the four cohorts of eggs (Y-Eu, Y-An, O-Eu, and O-An). There were no differences in GV area (Y-Eu; 432.18 ± 3.00 vs. Y-An; 419.91 ± 5.42 vs. O-Eu; 417.37 ± 5.55 vs. O-An; 427.45 ± 13.50 µm^2^), PVS area (Y-Eu; 801.94 ± 46.01 vs. Y-An; 1024.2 ± 159.90 vs. O-Eu; 897.50 ± 68.39 vs. O-An; 787.36 ± 60.81 µm^2^), ZP area (Y-Eu; 2060.91 ± 26.98 vs. Y-An; 2061.71 ± 45.78 vs. O-Eu; 1978.30 ± 38.14 vs. O-An; 1993.55 ± 43.29 µm^2^), PBI area (Y-Eu; 476.99 ± 11.76 vs. Y-An; 481.29 ± 31.11 vs. O-Eu; 469.38 ± 11.80 vs. O-An; 426.91 ± 33.79 µm^2^), and cytoplasm area (Y-Eu; 3790.65 ± 35.87 vs. Y-An; 3595.29 ± 62.61 vs. O-Eu; 3666.35 ± 45.28 vs. O-An; 3690.18 ± 74.59 µm^2^) among these groups of eggs (*p* > 0.05, Table 2, Fig. 5a, c-f). However, we observed a significant difference in the oocyte area among these four groups of eggs (Y-Eu; 4238.12 ± 37.13 vs. Y-An; 4035.29 ± 61.64 vs. O-Eu; 4083.72 ± 44.85 vs. O-An; 4117.64 ± 67.83 µm^2^) with the oocyte area in the O-Eu group being significantly smaller than in the Y-Eu group (*p* = 0.031, Table 2, Fig. 5b). We pooled the young and old oocytes together and classified them only based on the ploidy status. After this analysis, we observed no differences in the morphological parameters including GV area (428.20 ± 2.69 vs. 423.68 ± 7.14 µm^2^), oocyte area (4181.86 ± 29.40 vs. 4085.61 ± 47.65 µm^2^), PVS area (839.05 ± 38.77 vs. 870.94 ± 71.53 µm^2^), ZP area (2030.81 ± 22.28 vs. 2020.06 ± 32.03 µm^2^), PBI area (474.26 ± 8.62 vs. 448.06 ± 24.18 µm^2^), and cytoplasm area (3745.36 ± 28.56 vs. 3653.28 ± 51.61 µm^2^) between euploid and aneuploid eggs (*p* > 0.05, Fig. 5g-l).

### Kinetics of cumulus expansion is similar between euploid and aneuploid eggs regardless of age

**Fig. 6:**
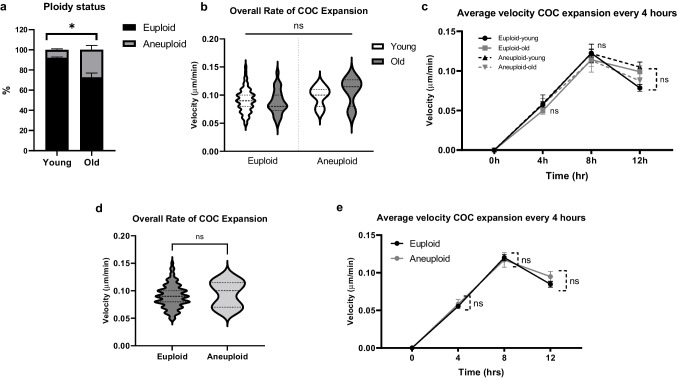
Morphokinetic parameters of cumulus expansion are similar between euploid and aneuploid eggs regardless of age. (**a**) Ploidy status of resulting MII eggs between young and old mouse COCs. (**b**-**c**) Morphokinetic parameters of cumulus expansion among 4 groups of eggs (Young-Euploid, Young-Aneuploid, Old-Euploid, and Old-Aneuploid) including (**b**) overall rate of cumulus expansion, (**c**) velocity of expansion every 4 h. (**d**-**e**) Morphokinetic parameters of cumulus expansion between euploid and aneuploid eggs (pooled young and old eggs) (**d**) overall rate of cumulus expansion and (**e**) velocity of expansion every 4 h. (Young; euploid *n* = 69 eggs, aneuploid = 5 eggs, Old; euploid *n* = 28 eggs, aneuploid *n* = 11 eggs, 3 replicates) (ns; *p* > 0.05) COCs; cumulus-oocyte complexes, MII; metaphase of meiosis II

Following IVM of intact COCs in EmbryoScope + ™, the incidence of euploid and aneuploid eggs from reproductively young mice was 92.31 ± 1.07% (*n* = 69) and 7.92 ± 0.91% (*n* = 5), whereas these numbers were 72.72 ± 4.49% (*n* = 28) and 27.36 ± 4.41% (*n* = 11) from reproductively old mice (Table 1, Fig. 3c). The incidence of euploid eggs was significantly decreased in COCs from reproductively old mice (*p* > 0.05, Table 1, Fig. 6a). We compared the morphokinetic parameters of cumulus expansion among the four groups of COCs (Y-Eu, Y-An, O-Eu, and O-An). There were no differences in the overall rate of cumulus layer expansion (Y-Eu; 0.091 ± 0.003 vs. Y-An; 0.099 ± 0.007 vs. O-Eu; 0.088 ± 0.004 vs. O-An; 0.108 ± 0.013 µm/min, *p* > 0.05, Table 2, Fig. 6b) among these groups of eggs. The velocity of cumulus expansion at every 1 h and every 4 h was also similar (*p* > 0.05, Fig. 6c, Supplemental Fig. 3b). The overall kinetics of expansion was similar in all groups, being faster during the first 8 h and then slower through the end of the maturation period. We pooled the oocytes together and classified them only based on ploidy status. After this analysis, we observed no differences in the morphokinetic parameters of cumulus layer expansion, including the overall rate of cumulus layer expansion (0.090 ± 0.002 vs. 0.094 ± 0.006 µm/min, *p* > 0.05, Fig. 6d) and the velocity of cumulus expansion at every 1 h and every 4 h between euploid and aneuploid eggs (*p* > 0.05, Fig. 6e, Supplemental Fig. 3c).

## Discussion

Reproductive aging occurs unequivocally in females and is associated with a gradual decrease in both gamete quantity and quality which leads to a progressive increase in infertility, miscarriage, and other health consequences [30]. Several functional and morphological alterations associated with reproductive aging include decreased ovarian reserve, ovulatory dysfunction, impaired ovulation, abnormal hormone production, altered extracellular matrix status, reduced ovarian wound healing, aberrant morphology of the ovarian surface epithelium, mitochondria dysfunction, abnormal meiotic spindle formation, chromosomal anomalies, granulosa cell apoptosis, decreased fertilization, as well as alterations in proteins associated cell cycle regulation and spindle machinery [31, 32, 40, 50–52]. Besides factors intrinsic to the oocyte, extrinsic factors associated with the oocyte microenvironment, mediated through cumulus cells follicular fluid, and the stroma, also play a role in the age-associated decline of oocyte quality [53]. These factors contribute to the decreased developmental competence of the associated gamete [32, 54].

This study extends state-of-the-art time-lapse technology (EmbryoScope + ™) and morphokinetic analysis used in clinical ART to evaluate the effect of physiologic reproductive aging on mouse oocyte IVM [3, 55]. We used oocytes and COCs from reproductively young and old mice to determine the effects of reproductive aging on this process. This model of physiological aging is validated and demonstrates a decrease in gamete quantity and quality [7]. The efficiency of meiotic maturation was similar in oocytes from both reproductively young and old mice, with 93–97% of the oocytes extruding the first polar body. This incidence of meiotic progression is similar to our previous study where we reported that approximately 91–96% of oocytes from reproductively young mice extruded a polar body in the EmbryoScope + ™ system [12]. This is on average higher than other studies in traditional incubators where the maturation success is ~ 75–90% [7, 31, 49, 56]. The incidence of egg aneuploidy increased from 8–9% in reproductively young mice to 24–29% in reproductively old mice, which is consistent with previous studies which found a higher aneuploidy incidence with advanced reproductive age in in vitro matured MII eggs [7, 56, 57]. The aneuploidy incidence of eggs in our study is also consistent with that observed in vivo (~ 3–10% for eggs from reproductive young and ~ 25–35% for reproductively old mice), which provides support that the EmbryoScope + ™ mirrors physiologic conditions [49, 56–59]

Morphokinetic parameters of meiotic progression including time to GVBD, time to PBE, and duration of meiosis I in reproductively young oocytes were similar to the findings in our previous study [12]. However, these morphokinetic parameters were shorter, approximately 0.75 – 1.5 h, than those reported in previous studies in which oocytes were matured in conventional time-lapse incubators [7, 60, 61]. One study reported that the oocytes started PBI extrusion at 8 h after the onset of IVM, but the majority of oocytes extruded PBIs after 14 h [40]. Additionally, we did not observe any differences in all morphokinetic parameters of meiotic progression between reproductively young and old mouse oocytes. Our findings are consistent with a previously published study that analyzed meiotic progression in individual oocytes from reproductively young and old mice in a conventional time-lapse chamber and did not demonstrate any differences in time to GVBD, time to PBE, and duration of meiosis I [7]. In contrast, another study revealed that oocytes from aged CBA/Ca mice progress through the first meiotic division approximately 1.5 h faster compared to oocytes from young counterparts [62]. These discrepancies could be due to different IVM systems used [63] and the inherent biological differences between mouse strains [7, 38, 61, 64]. Furthermore, the consistent maintenance of optimal temperature and gas concentrations in EmbryoScope + ™ provides a more stable culture environment than those in traditional time-lapse systems and might better phenocopy events in vivo [5, 65].

Our results demonstrate that the GV and oocyte area are significantly smaller in the reproductively old mouse oocytes. Although there were no significant differences in the other morphological parameters, the PBI area, ZP area, and cytoplasm area tended to be smaller and the PVS area larger with advanced age. This is consistent with a previous study which demonstrated that the cytoplasm diameter and ZP thickness linearly decrease, whereas the PVS area increases with advancing maternal age [40, 66]. In contrast, another study demonstrated no age-dependent differences in the proportion of morphologically normal eggs in reproductively young and old mice [50]. There was no strong correlation among these morphological parameters in oocytes either from reproductively young or old mice except for oocyte and cytoplasm area, which is similar to our previous study [12]. Furthermore, no correlations were observed between oocyte morphological and morphokinetic parameters in either age group.

During IVM, the cumulus cells exhibited dynamic behavior, with expansion velocity occurring faster and peaking during the first 8 h of IVM compared to the later periods in both reproductively young and old groups, which is consistent with our previous observations of cumulus expansion in the EmbryoScope + ™ [12], [Dipali et al., Biology of Reproduction, under review]. Previous studies have demonstrated that the genes involved in the expansion process, including hyaluronan synthase 2 (*Has2*), prostaglandin endoperoxide synthase 1, 2 (*Ptgs1, Ptgs2*), and tumor necrosis factor-alpha-induced protein 6 (*Tnfaip6*) are highly expressed at 4—8 h post-IVM or ovulation induction, and then their levels gradually decrease [67, 68]. Our findings also support the work which demonstrated that the invasive potential of cumulus cells increases steadily and reaches a peak at ovulation [69]. Although we did not observe any significant differences in kinetics of cumulus expansion between oocytes from reproductively young and old mice, the overall rate of cumulus expansion was slightly slower with advanced reproductive age (0.093 ± 0.002 vs. 0.089 ± 0.003 µm/min). There were also different trends in the pattern of cumulus expansion, whereby COCs from reproductively young mice expanded faster early (0-8 h) in culture and slowed down more rapidly, whereas COCs from reproductively old mice appeared slightly delayed. Cumulus cell biology along with intercellular communication between the oocyte and somatic cells appears altered with age [53, 70]. Apoptosis in cumulus and granulosa cells linearly increases with age and is associated with poor reproductive outcomes in humans [54, 71]. It is possible that intrinsic differences and/or altered responses to hormones and growth factors may underlie age-dependent trends in cumulus cell behavior. Given the essential role of cumulus cells in ovulation and increased rate of ovulation abnormalities in older mice [50], studies are ongoing to elucidate comprehensive differences in cumulus cells between reproductively young and old mice.

Egg aneuploidy increases in mouse and human with advanced reproductive age due to numerous factors, including recombination defects, weakened chromosome cohesion, altered chromosome micromechanics, and age-associated spindle dysfunction during oocyte meiosis [17, 49, 72–74]. We did not observe any differences in morphokinetic parameters of meiotic progression or cumulus layer expansion between euploid or aneuploid eggs irrespective of age. This is consistent with previous reports that did not demonstrate an association between morphokinetics parameters of meiotic progression, timing of anaphase I onset, cumulus layer expansion, and oocyte ploidy [7, 12]. Our findings are also indirectly supported by a study which compared the duration of meiosis I between control eggs and those in which chromosome misalignment was induced where no differences in the duration of meiosis I were observed [61]. Furthermore, previous studies observed similar rates and timing of GVBD, in mouse oocytes harboring DNA damage during meiosis I [75, 76].

The link between morphokinetics of cell division and aneuploidy has also been investigated in the context of human preimplantation embryo development where the results are conflicting. Some morphokinetic studies in the human preimplantation embryo were unable to identify any significant difference in time-lapse parameters between euploid and aneuploid embryos [77–79]. Conversely, other studies showed a significant correlation between morphokinetic parameters of embryo development (e.g. time to pronuclear fading (tPNf), time to 2 cells (t2), time to 5 cells (t5), and time to blastulation) and ploidy status [80–84]. These results underscore the importance of further studies in this area.

We used a physiological reproductive aging mouse model and a tightly controlled optimal culture environment with the EmbryoScope + ™ to track oocytes and COCs individually and rigorously correlate the time-lapse features with age and ploidy status. It is possible that our system may not fully recapitulate in vivo oocyte maturation because reproductively young and old mice were hyperstimulated with exogenous gonadotropins, and IVM occurred in the absence of the entire follicle. However, we used a routine hyperstimulation protocol involving PMSG for both reproductively young and old cohorts of mice. In our previous study, we demonstrated that both age cohorts respond similarly in terms of endocrine response to this stimulation protocol [50]. Furthermore, we have used specific media that is optimized for either spontaneous meiotic maturation or intact COCs as evidenced by our high maturation rates and low aneuploidy rates [12]. Importantly, samples from reproductively young and old mice were matured in parallel and treated similarly under the same conditions to minimize any intervention bias, so the media composition should not obscure any age effects. Moreover, the incidence of aneuploidy was consistent with what is observed in in vivo-matured eggs in this strain of mice [56].

The goals of IVM for humans are 1) to increase the number of mature eggs for infertility treatment and 2) increase the number of mature eggs banked for fertility preservation [85]. Cumulus cells which surround the oocyte are essential for generating a high-quality gamete by providing nutrients and enabling metabolic cooperativity [86, 87]. In fact, outcomes of human oocyte IVM are significantly better in the presence of cumulus cells [88]. Time-lapse imaging of COCs and the assessment of cumulus expansion velocity along with other baseline parameters reported in this study could be utilized in human IVM and correlated to embryo development and pregnancy outcomes. Such knowledge may help define optimal IVM parameters and enable the selection and prioritization of embryos for transfer. Furthermore, these parameters could serve as a foundation for testing different conditions such as supplements, media, and culture conditions to improve human IVM outcomes. However, human COCs are larger than mice, so the field of view in the current EmbryoScope + ™systems are not sufficient to monitor human cumulus cell expansion. Thus, other time-lapse technologies may need to be developed for this purpose.

In conclusion, we did not observe robust differences in the morphokinetic parameters of oocyte maturation, including meiotic progression and cumulus expansion, in regards to age and ploidy status in the mouse model. Whether this holds true for human IVM warrants further investigation. Of note, the quality and developmental potential of the egg are dictated by both meiotic and cytoplasmic competence as well as the microenvironment surrounding the oocyte [53, 89, 90]. Future studies to determine how these morphological and morphokinetic parameters correlate with fertilization and preimplantation embryo development are needed to better understand the predictive value of such information. Live time-lapse imaging integrated with artificial intelligence (AI) to analyze a large amount of acquired visual material and morphokinetic data will likely improve the predictive value of this technology to ultimately develop and apply the non-invasive assessment of gamete and embryos in the clinical setting [91]. A better understanding of oocyte maturation dynamics with these technologies will likely lead to the advancements in human IVM, which can ultimately improve outcomes for infertility treatment and provide alternative fertility preservation options to patients.

## Supplementary Information

Below is the link to the electronic supplementary material.

Supplementary file1 (PDF 159 KB)

Supplementary file2 (PDF 144 KB)

Supplementary file3 (PDF 67 KB)

Supplementary file4 (MP4 12759 KB)

Supplementary file5 (MP4 34395 KB)

## Data Availability

All original data in this publication are available upon reasonable request to the corresponding author.
