# Supplementary figures and images for: Morphokinetic parameters of mouse oocyte meiotic maturation and cumulus expansion are not affected by reproductive age or ploidy status
Source: J Assist Reprod Genet. 2023 Apr 4;40(5):1197–213. doi: 10.1007/s10815-023-02779-y (PMC10239409; doi:10.1007/s10815-023-02779-y)

Supplemental Fig. 1

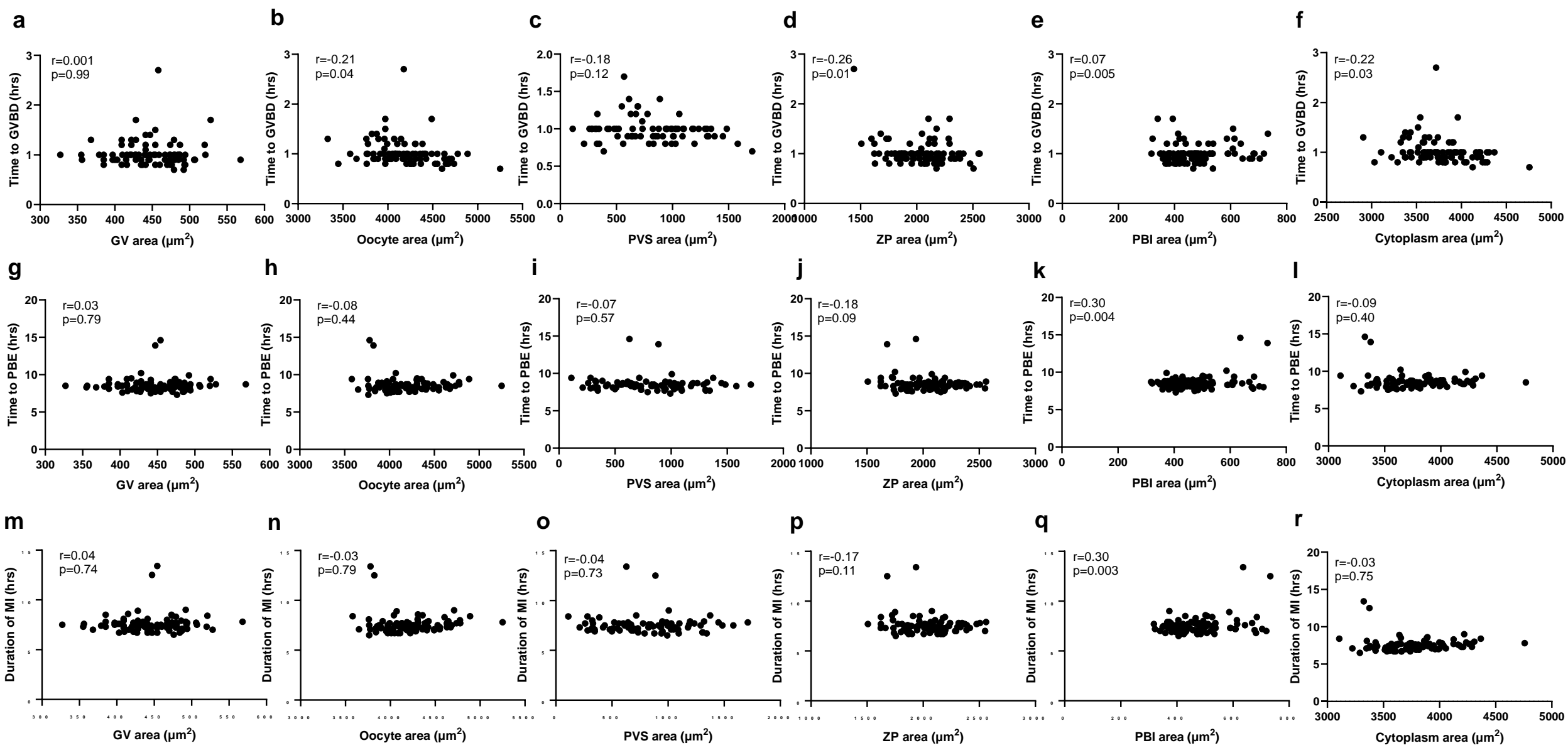

Supplement: Supplementary file 1 — Supplementary file1 (PDF 159 KB) [file 10815_2023_2779_MOESM1_ESM.pdf]

Supplemental Fig. 2

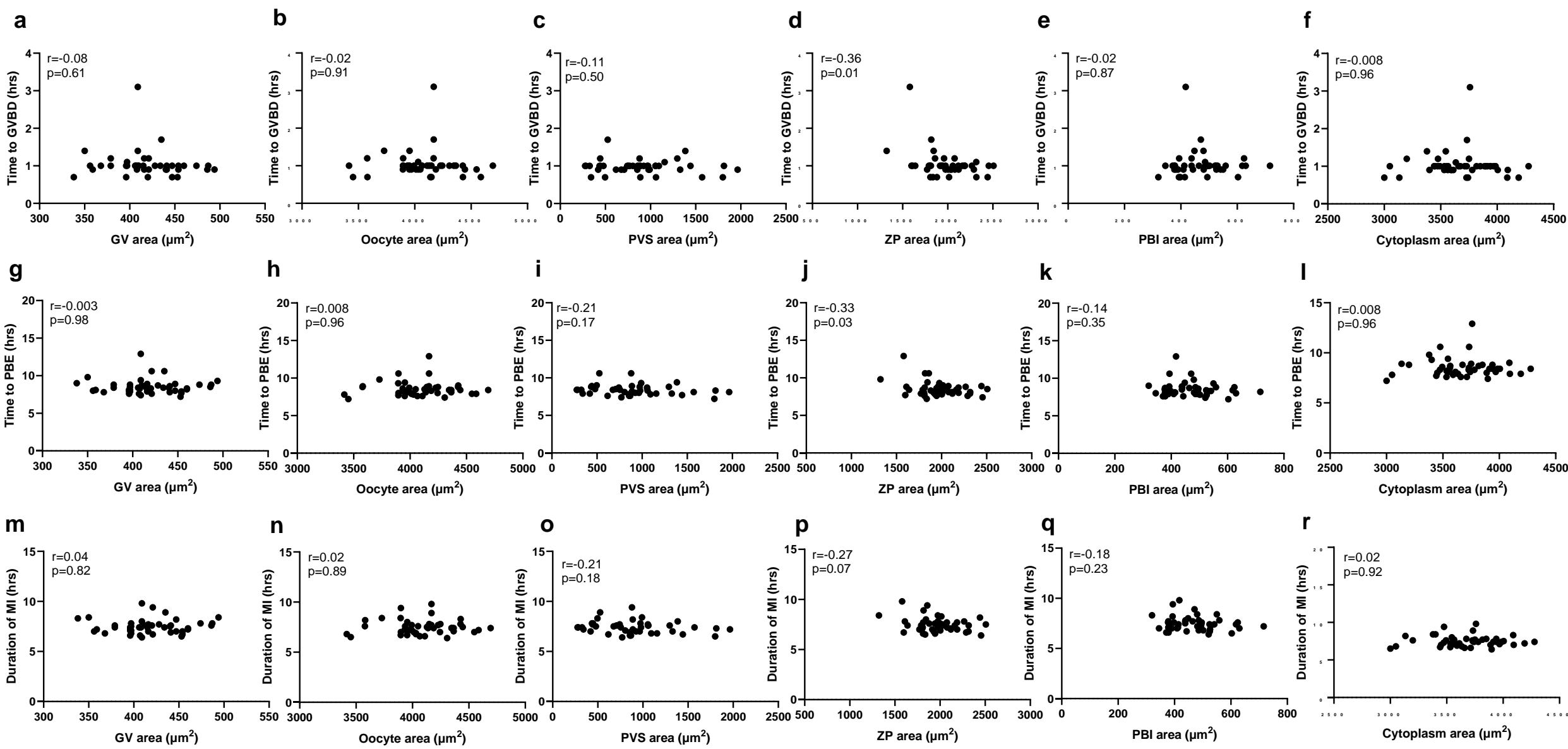

Supplement: Supplementary file 2 — Supplementary file2 (PDF 144 KB) [file 10815_2023_2779_MOESM2_ESM.pdf]

Supplemental Fig. 3

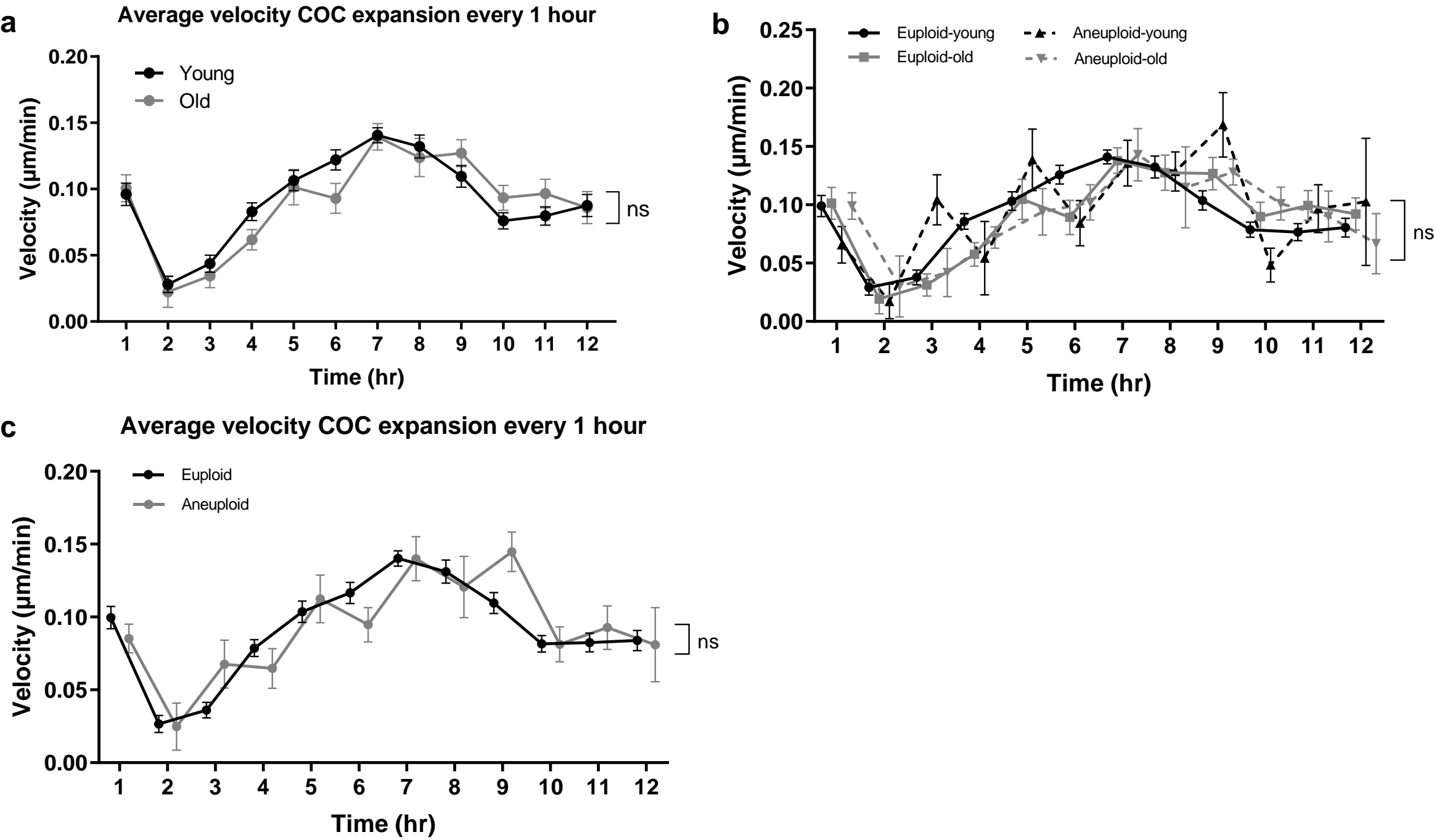

Supplement: Supplementary file 3 — Supplementary file3 (PDF 67 KB) [file 10815_2023_2779_MOESM3_ESM.pdf]
